# Emulsions stabilized by cellulose-based nanoparticles for curcumin encapsulations: *In vitro* antioxidant properties

**DOI:** 10.3389/fnut.2022.931581

**Published:** 2022-07-22

**Authors:** Jian Zhang, Huien Zhang, Liping Liu, Zhongfa Chen

**Affiliations:** Zhejiang Provincial Top Discipline of Biological Engineering (Level A), College of Biological and Environmental Sciences, Zhejiang Wanli University, Ningbo, China

**Keywords:** different cellulose structure, surface element, curcumin emulsion, stability, antioxidant properties

## Abstract

To improve the dispersity and antioxidant properties of curcumin, curcumin emulsions covered with cellulose particles (CP) with different structures were successfully prepared, and the structural characteristics, stability, and antioxidant properties of emulsions were investigated. The results showed that the CP obtained by increasing the hydrolysis time had smaller particle size, better water dispersion, and interfacial adsorption capacity. The encapsulation efficiency of curcumin in emulsion stabilized by cellulose particle hydrolyzed for 10 h can reach about 80%. After 9 days, all emulsions showed good stability, and no obvious creamed layer was observed. Compared to cellulose particles hydrolyzed for 2 h (CP2), emulsions stabilized by cellulose particles hydrolyzed for 6 h (CP6) and 10 h (CP10) exhibited better stability and free fatty acid (FFA) release. Meanwhile, the DPPH scavenging activity of curcumin emulsion stabilized by CP significantly increased with increasing the hydrolysis time and was much higher than that of pure emulsion and curcumin/water due to the higher solubility (1,455 times compared with curcumin/water solution) of curcumin, and these results could provide useful data for the stability and encapsulation of curcumin.

## Introduction

Bioactive substances, such as curcumin, frequently need to be protected from environmental conditions like temperature, light, pH, and oxygen due to their chemical and physical instability (1). To increase the stability and bioavailability of bioactive substances, designing a stable oil-in-water emulsion transport carrier was the common solution. Emulsions stabilized by natural macromolecules, e.g., soy protein, whey protein, Arabic gum, modified starch, and flavonoids, have been confirmed to exhibit more sustained release of some encapsulated lipophilic ingredients (2, 3), as compared with conventional surfactant-based emulsions. This implies that these kinds of emulsions can be developed into effective delivery systems with good functional performance, thus exhibiting a good potential for the development of novel functional foods (4, 5). As the world's population grows, the demand for food increases dramatically. According to the United Nations, the world's population will grow to 9 billion by 2050 (6). Therefore, some edible good proteins, carbohydrates, and other resources will become scarce resources. At the same time, with the enhancement of people's awareness of environmental protection, renewable and environmentally friendly materials will become the focus of industrial development. However, cellulose has greater advantages in biocompatibility, biodegradability, high strength, strong surface activity than other natural resources (7, 8). Meanwhile, cellulose is the most abundant natural resource on earth with an annual global output of 1.5 × 10^12^ tons (9). The main sources of cellulose are wood and cotton. For centuries, these materials have been used as heat sources or building materials, or in the manufacture of several commodities in the textile and paper industries. Due to its insolubility, hygroscopicity, and non-melting properties in water and most organic solvents, the use of cellulose in high-value-added applications is still rare (10).

Several researchers have done a lot of work on cellulose (cellulose nanofibrils, cellulose microfibrils, cellulose nanocrystals, bacterial cellulose, etc.) that could be self-assembled at the oil–water interface and stabilize O/W emulsion without the addition of an emulsifier. Capron et al. (11) found that the addition of <0.1% (w/w) of cellulose sulfate nanocrystals was sufficient to form a stable high internal phase O/W emulsion with a volume fraction of 0.9%. Kargar et al. (12) reported that the mechanical barrier against coalescence was obtained by forming a network of cellulose around the emulsion droplets. The non-adsorbed particles act as thickeners in the continuous aqueous phase. Chen et al. (13) prepared stable high internal phase emulsions using OSA-modified cellulose nanocrystals. The average particle size of the emulsion in the previous literature was several microns or even a dozen microns, and the emulsion also showed different degrees of instability. The adsorption properties of cellulose at the interface are greatly affected by its shape, size, aspect ratio, and surface structures. However, whether nanoscale cellulose particles can form a more stable emulsion and the adsorption mechanism of cellulose particles with different surface structures at the emulsion interface are still unclear.

Thus, the curcumin emulsions covered with CP with different structures were successfully prepared, and the structural characteristics, stability, and antioxidant properties of the emulsions were investigated. The relationship between CP and the interfacial adsorption properties and antioxidant properties of emulsion were analyzed. The purpose was to reveal the relationship between the interfacial adsorption capacity and the oxidation resistance of CP. These results could provide useful data for the stability and encapsulation of curcumin.

## Materials and methods

### Materials

Curcumin (98% pure) was purchased from Sigma Aldrich. Cellulose and medium-chain triglycerides (MCT) were purchased from Sinopharm Chemical Reagent Co., Ltd. (Shanghai, China). All chemical reagents used in this study were of reagent grade. All water used in the experiment was ultrapure obtained from a Millipore Direct-Q 5UV apparatus (Merck, Darmstadt, Germany).

### Nanocellulose particle preparation

Nanocellulose was prepared according to our previously reported method with minor modifications (14). A total of 5 g of microcrystalline cellulose was weighed in a beaker and then 60 ml of mixed acid (30% sulfuric acid and 15% hydrochloric acid) and 40 ml of distilled water were added for hydrolysis. After hydrolysis for 2, 6, and 10 h, the sample was centrifuged to remove the acid and then repeated washing was done with distilled water until neutral. Samples were sealed and stored at 4°C until further analysis. The cellulose particle obtained by hydrolysis at 2, 6, and 10 h were marked as CP2, CP6, and CP10, respectively.

### Surface element analysis

The surface element composition of CP was carried out by X-ray Photoelectron Spectroscopy (XPS) (Thermo ESCALAB 250XI, USA). The spectrometer was equipped with a monochromatic Al Kα (*hv* = 1486.6 eV) X-ray source of 150 W at 15 kV. The kinetic energy of photoelectrons was determined with a hemispheric analyzer set to pass energy of 160 eV for wide-scan spectra and 20 eV for high-resolution spectra, respectively. During all measurements, electrostatic charging of the samples was avoided by means of a low-energy electron source working in combination with a magnetic immersion lens. Later, all recorded peaks were shifted by the same value to set the C1s peak to 284.8 eV. Quantitative elemental compositions were determined from peak areas using sensitivity factors experimentally and the spectrometer transmission function. The high-resolved spectra were deconvoluted using a computer routine. Peaks were fitted to Gaussian–Lorentzian ratio (8:2) curves after subtraction of baseline.

### Contact angle measurement

Contact angles of the distilled water on CP film were measured at 25°C in an ambient atmosphere with a contact angle goniometer (Drop shape Analyzer DSA25, Kruss, Germany). The image was then recorded with a video camera immediately after dripping water. A Laplace-Young fit. 2.4 was applied to construct the contour of the imaged droplets, and the contact angles were determined.

### Emulsion preparation

A total of 0.2 wt% of curcumin was added into MCT at 60°C, stirred for 10 min, and then ultrasonic treatment was given for 20 min to obtain the oil phase. Crude emulsion was prepared by homogenizing 1 ml oil phase and 99 ml 0.5 wt% CP dispersion using an Ultra-Turrax blender (IKA T25 Basic, Staufen, Germany) at 11,000 rpm for 2 min. Fine emulsions were prepared by twice homogenization at 40 MPa using the homogenizer (APV1000, APV Co., Crawley, U.K.). After preparation, all emulsions were stored at 4°C.

### Particle size and ζ-potential measurements

The particle size of the emulsion and CP were measured by dynamic light scattering (Zetasizer Nano ZS, Malvern instruments, UK), and each individual measurement was averaged 12 times. To avoid the effect of multiple scattering, buffer solution (5 mM phosphate, pH 7) was used to dilute the emulsion before analysis.

Zetasizer Nano ZS (Malvern Instruments Ltd., Malvern, UK) was used to measure the ζ-potential of the emulsion during storage for 9 days at 25°C. All emulsion samples were fresh and stable. ζ-Potential was reported as the average value and standard deviation of the three newly prepared samples. Three readings for each sample was taken as the average value.

### Morphology observation

To observe the microstructure of CP and emulsion stabilized by CP, an Olympus BX 51 optical microscope equipped with a digital camera (Olympus, DP 50) was taken. At room temperature, the sample was uniformly injected onto the microscope slide and observed with 10× and 40× magnification.

### *In vitro* digestion

The release kinetics of free fatty acid (FFA) in emulsions during *in vitro* digestion was evaluated using a simulated intestinal fluid (SIF). A total of 10 ml of each emulsion was transferred to a clear amber bottle and then 100 ml of SIF was added. The mixture was then incubated for 2 h at 37°C with simulated small intestinal fluid (SIF) containing 1 ml calcium chloride solution (750 mM),4 ml bile extract solution (5 mg/ml), and 2.5 ml pancreatic lipase (4.8 mg/ml). After mixing the solution, the pH was stabilized at 7.0 by sodium hydroxide. The amount of alkali solution (0.5 M NaOH) used to maintain pH 7.0 to determine the proportion of FFA released by the system was recorded. The control group experiment was carried out under the same conditions. The released FFA was calculated after subtracting the amount of alkali consumed in the control group reaction from the sample. After digestion for 2 h, the samples were collected for physical, chemical, and structural characterization.

### Encapsulation efficiency determination of curcumin

The amount of curcumin entrapped in the emulsion was determined spectrophotometrically (UV-2600, Shimadzu, Japan) at 425 nm. For each type of sample, 50 μl was dissolved in 4 ml of 95 ml/100 ml of acetonitrile aqueous solution and mixed for 48 h to ensure the embedded curcumin can be dissolved into the solution. The solution was centrifuged at 10,000*g* for 10 min and then passed through a 0.22-μm nylon filter to remove all insoluble substances in the solution. Calculations were done using the following equation: A = 0.1438C−0.0073 (R^2^ = 0.99), where A and C are the absorbance and concentration of curcumin in anhydrous ethanol, respectively. R^2^ was the correlation coefficient of the linear regression equation. Encapsulation (EE) of curcumin in the emulsion was determined by the following equation


(1) $$\begin{array}{l} \begin{array}{cc} EE(\%)\;=\; & (\text{total curcumin}-\text{free curcumin})/ \end{array}\\ \;\begin{array}{c} \text{ total curcumin(mg})\;\times \;100\%. \end{array} \end{array}$$

### Antioxidant properties of curcumin emulsion

The DPPH radical scavenging activity of the sample was determined using the method described in the literature (15, 16). The DPPH working solution was prepared by dissolving the DPPH reagent in ethanol with a concentration of 0.1 mm. A total of 1 mL curcumin ethanol solution or sample aqueous solution was added to 4 ml of the above working solution. The mixed solution was shaken evenly and left to stand in the dark at room temperature for 30 min. The absorption was recorded at 517 nm on the UV-Vis spectrophotometer. The DPPH radical scavenging activity was estimated according to the following equation:


(2) $$\begin{array}{l} \begin{array}{cc} \text{DPPH radical scavenging activity}\;(\%)\;=\; & (A_{\text{blank}}-A_{\text{sample}})/ \end{array}\\ \;\begin{array}{c} A_{\text{blank}}\;\times \;100, \end{array} \end{array}$$

where A_blank_ and A_sample_ are the absorption of DPPH solution added with distilled water and sample solution respectively at 517 nm. All tests were tested three times in parallel, and the results were expressed as mean and standard deviation.

### Statistical analysis

Statistical analysis of the data was carried out using one-way analyses of variance (ANOVA), and significant differences (*p* < 0.05) between the samples were determined by Duncan's multiple range procedures using SPSS software (version 19, IBM software, NY, USA). Each experiment was replicated at least three times.

## Results and discussion

### Effects of hydrolysis time on the surface element and water dispersibility of CP

**Figure 1:**
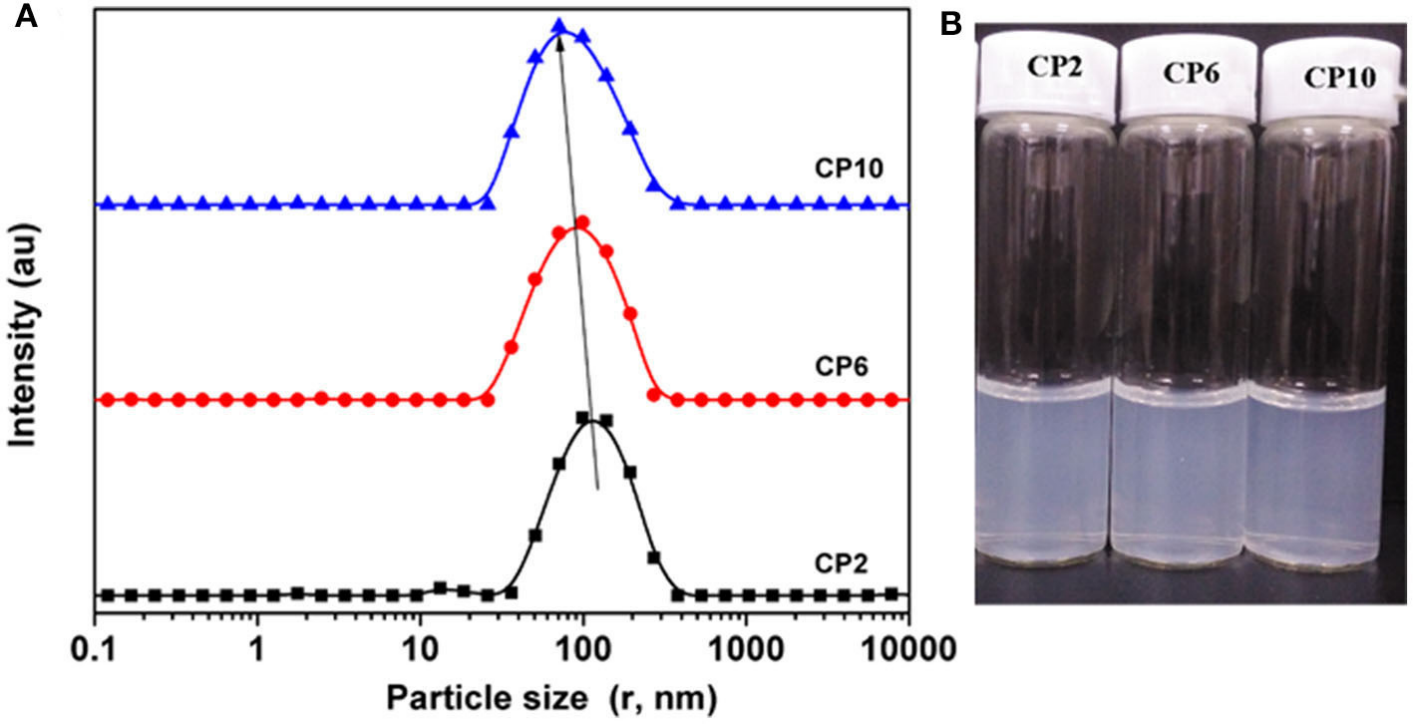
The particle size distributions **(A)** and water dispersibility **(B)** of CP at different hydrolysis times.

**Figure 2:**
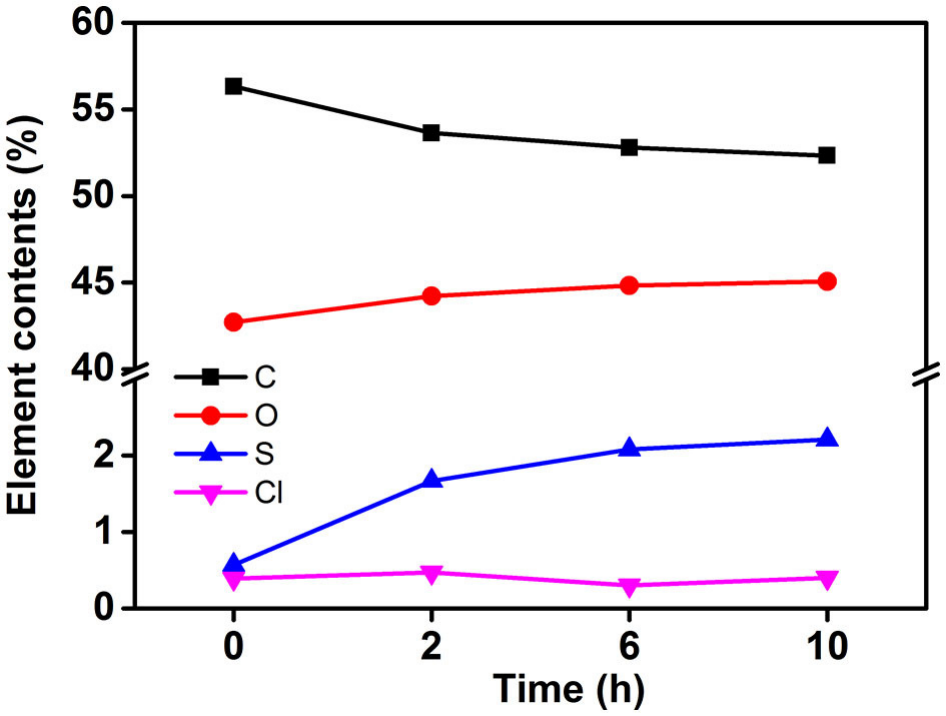
Percentage atomic content and oxygen–carbon ratio of each element on the surface of cellulose prepared at different hydrolysis time.

To obtain stable cellulose particles, the mixed acid hydrolysis method was used to prepare CP. The particle size distribution of CP with different prepared structures was studied. When the hydrolysis time was 2 h, the PSD curve showed two peaks, and the peaks appeared between 100 and 200 nm. On increasing the hydrolysis time, the PSD shifted toward a smaller particle size. Meanwhile, small peaks (10–20 nm) also moved to lower values. This may be due to further decomposition of previously hydrolyzed fragments. When the hydrolysis time reached 10 h, the particle size of CP further decreased and formed nanoparticles. Figure 1B shows the water dispersibility of CP. On the whole, all the three particles had certain turbidity, which indicated that there were particles with different sizes in the dispersion. But there was no significant difference between the three dispersions. At the same time, the three dispersions showed excellent stability and no precipitation occurred after a month of storage. This was mainly due to the change of elemental composition on the surface of CP with the increase of hydrolysis time, which made the particles carry more charge. The carbon content on the CP surface decreased and the oxygen content increased on increasing the hydrolysis time (Figure 2). This may be caused by two reasons: (i) more lignin was removed by increasing the hydrolysis time. Lignin was a complex phenolic polymer composed of three alcohol monomers: coumarinol, coniferol, and glucosamine, whose molecular structure oxygen/carbon ratio was smaller than that of cellulose; (ii) -C-${\text{SO}}_{3}^{-}$ and -O-${\text{SO}}_{3}^{-}$ groups were formed by esterification or sulfonation reaction between sulfuric acid and hydrocarbon or alcohol groups on the surface of cellulose. After the partial hydrogen element was removed, the content of the oxygen element increased, resulting in the increase of final O/C content. Meanwhile, it was obvious that the content of the sulfur element significantly increased, while the content of the Cl element did not change significantly. This result further proved that the higher O/C ratio of cellulose was due to more H-${\text{SO}}_{3}^{-}$ binding to the cellulose surface. At the same time, more H-${\text{SO}}_{3}^{-}$ bonded to the surface of cellulose could increase the charge of cellulose and then increase the electrostatic repulsion between cellulose particles, which was conducive to the stability of cellulose suspension.

**Figure 3:**
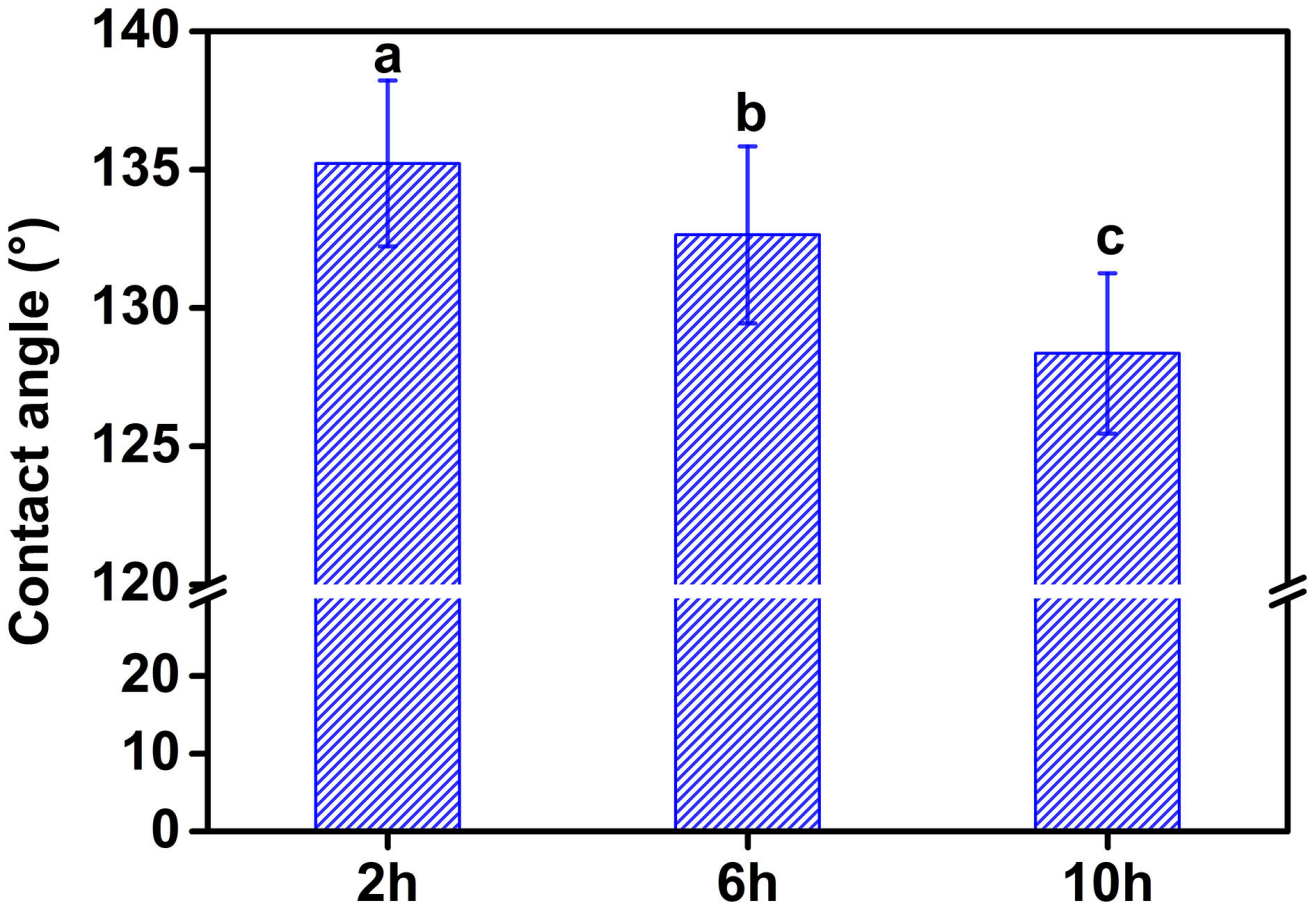
Contact angle of water droplets attached to cellulose films prepared under different hydrolysis time. Different letters (a–c) represent significant difference at *p* > 0.05 level between different samples.

By increasing the hydrolysis time, the contact angle of the CP significantly decreased (Figure 3). This result indicated that CP10 was more favorable to adsorb on the surface of oil droplets due to better water dispersion. The suitable wetting condition for the particle to effectively act as a stabilizer is only when θ is close to 90° (17). Therefore, the CP obtained by increasing the hydrolysis time had a smaller particle size, better water dispersion and interfacial adsorption capacity.

### Effect of hydrolysis time on the mean particle diameter of curcumin emulsion with storage time

**Figure 4:**
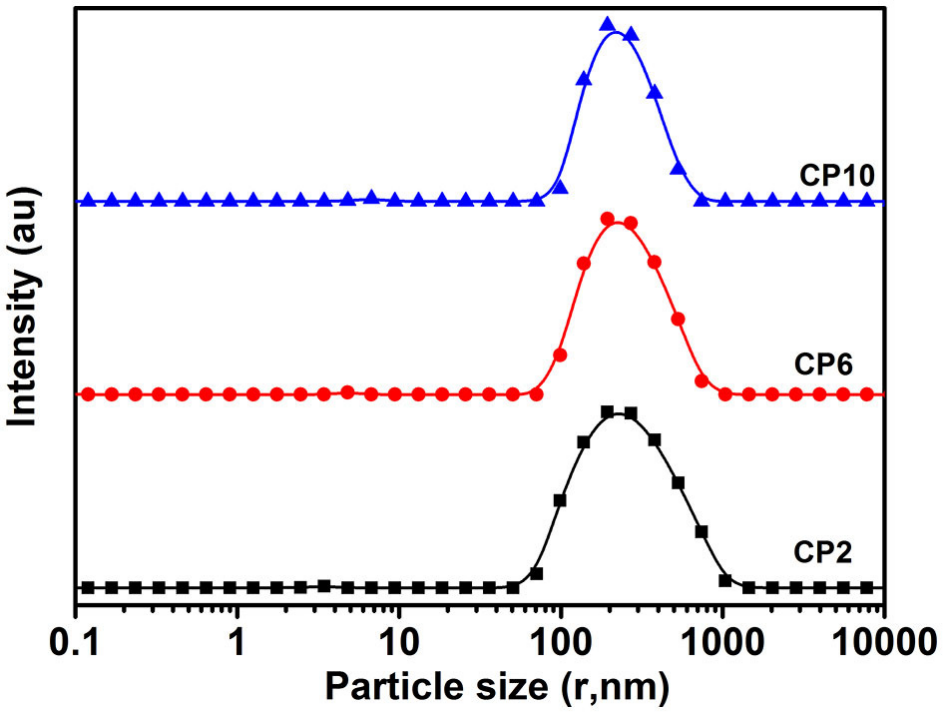
The particle size distributions of curcumin emulsion stabilized by CP. Different characters represent the significant difference at *p* < 0.05 level (*n* = 3).

**Figure 5:**
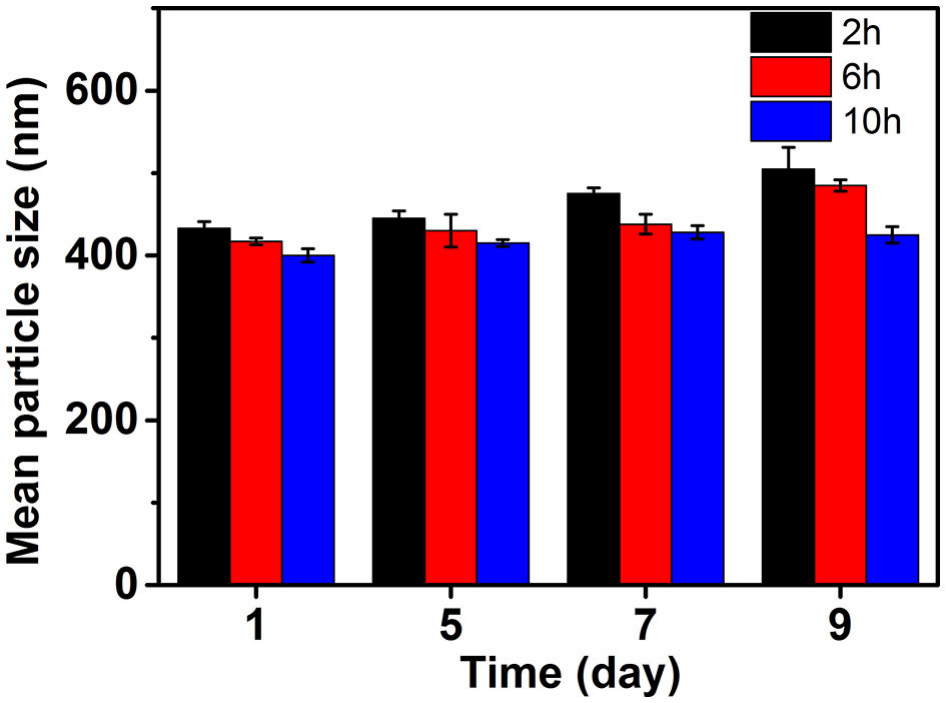
Mean particle size changes of curcumin emulsion stabilized by CP during 9 days' storage period at 25°C.

**Figure 6:**
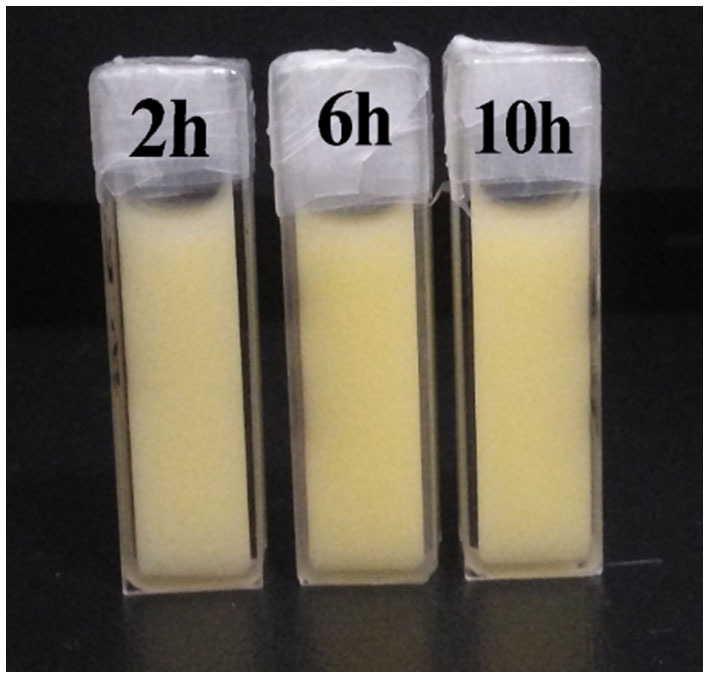
Images of curcumin emulsions stabilized by CP prepared at different hydrolysis times during storage for 9 days at 25°C.

The PSD curve of curcumin emulsion stabilized by different CPs is shown in Figure 4. Almost all the PSD curves were distributed in the range of 50–2000 nm. Interestingly, emulsions stabilized by CP2 had the widest particle size distribution. On decreasing the cellulose particle size, PSD became narrower. This was mainly because there was a positive correlation between emulsion particle size and emulsifier particle size (18). To evaluate the effect of hydrolysis time on the mean particle size of curcumin emulsion with storage time, the droplet size and digital pictures of the emulsions stabilized by CP were investigated under different hydrolysis times. Figure 5 shows the mean particle size emulsion stored for 9 days at 25°C. With the increase in hydrolysis time, the particle size of emulsion stabilized by CP significantly decreased after one-day storage. These results showed that the smaller the CP was, the smaller the droplet size of the emulsion was. In other words, the size of the emulsifier affected the relationship between the particles and particle-stabilized emulsions. With the increase in hydrolysis time, the particle size of CP decreased. The curcumin emulsion with different hydrolysis time also showed a similar trend. When the hydrolysis time was 2 h, the mean particle size of the emulsion was larger. The mean particle size of the emulsion increased with the increase in storage time (Figure 1). The particle size of CP was also reduced to <200 nm. When CP was larger, the adsorption state of CP on the surface of oil droplets was relatively looser. As the CP size decreases, the curcumin emulsion also became more and more stable. The reason was that more CP formed an adsorption layer at the droplet interface with the extension of hydrolysis time. This indicated that if the concentration of the control particles was constant, the emulsion size of the emulsifier particles is positively correlated with the size of the emulsifier particles. Binks et al. (19) found that the emulsions stabilized by bacterial cellulose nanocrystals (259.6 nm) revealed more uniform and smaller droplets in comparison with that stabilized by bacterial cellulose (483.6 nm) at the same concentration, indicating that bacterial cellulose nanocrystals possessed better emulsifying performance than bacterial cellulose. After storage for 9 days, all the emulsions showed good stability (Figure 6). Although the particle size of curcumin emulsion stabilized by CP was different after 9 days, the creaming phenomenon could be further inhibited. This may be because CP forms a thick interface layer on the surface of oil droplets, reducing Ostwald ripening of oil droplets.

### Effect of hydrolysis time on the surface charge of curcumin emulsion

**Figure 7:**
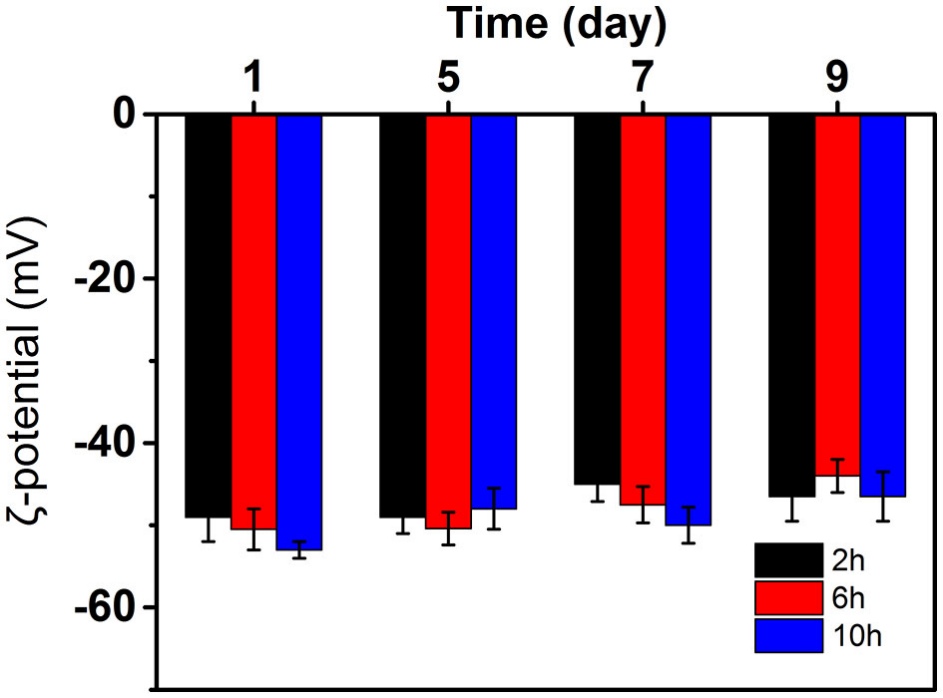
ζ-Potential of curcumin emulsions stabilized by CP prepared at different hydrolysis time during storage for 9 days at 25°C.

**Figure 8:**
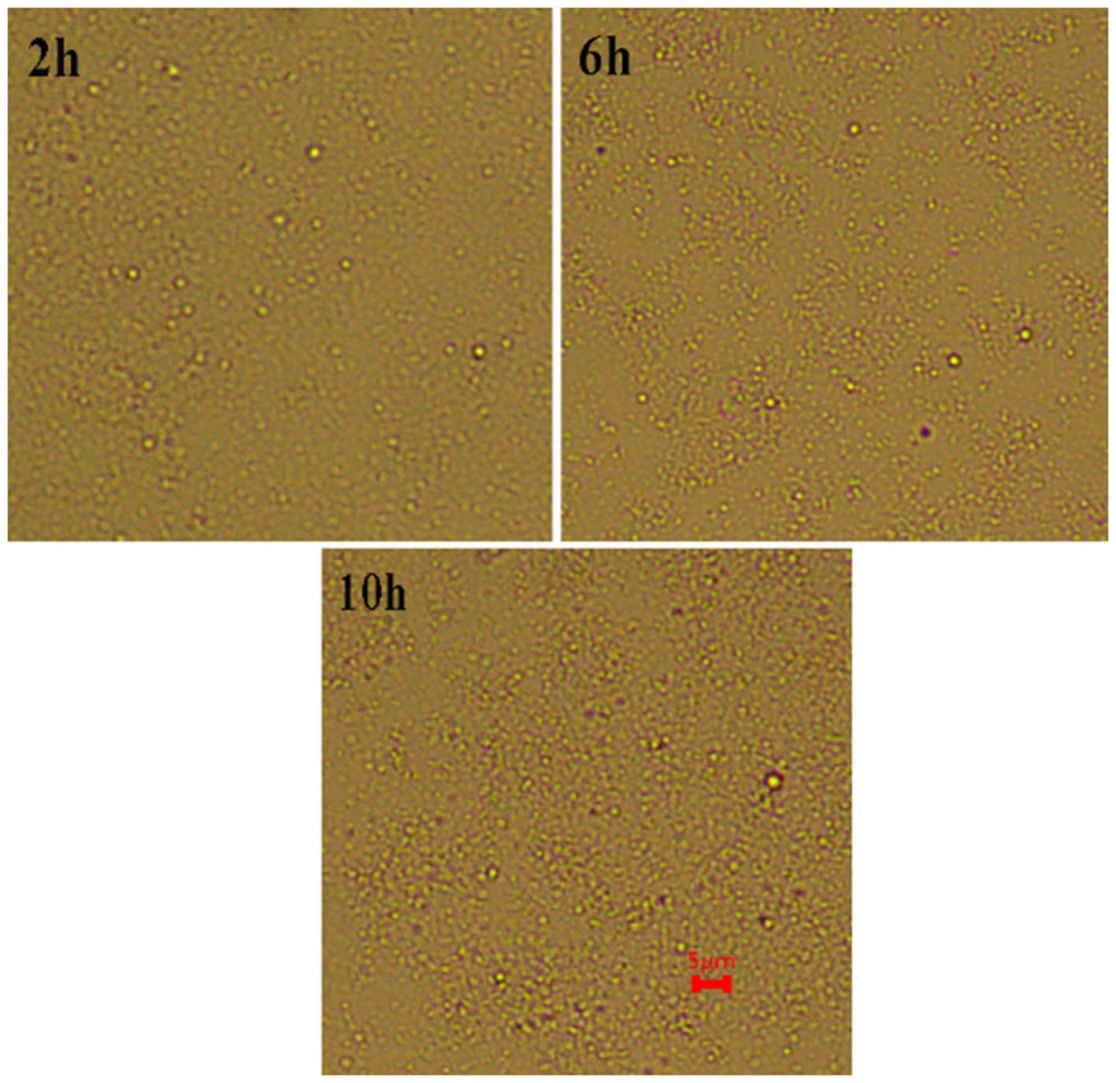
Microstructure of curcumin emulsions stabilized by CP prepared at different hydrolysis time during storage for 9 days at 25°C. The scale bar is 5 μm.

The ζ-Potential test is a simple method that can be used to evaluate the role of the electrostatic force in the stabilization mechanism. Generally, 30 mV is used to stabilize the boundary between emulsion and unstable emulsion (20). A higher absolute potential value means that the system has good stability. When ζ-potential was low, it was often accompanied by aggregation, flocculation, or coalescence. The ζ-potential of curcumin emulsion stabilized by CP prepared at different hydrolysis times are presented in Figure 7. The ζ-potentials of three emulsions reached −50 mV on the first day of storage, suggesting that the curcumin emulsion had potential stability. The ζ-potential of emulsion decreased significantly with the prolongation of hydrolysis time. This was mainly because CP dispersion had a large number of negative charges due to more O-${\text{SO}}_{3}^{-}$, C-SO_3_^−^, and C-OH groups after the hydrolysis and improved the stability of the curcumin emulsions (21). As the storage time increased, the slight decrease in surface charge was because the CP rearranged on the surface of oil droplets through hydrophobic and electrostatic effects to better combine with oil droplets. Meanwhile, the aggregation and flocculation caused by Ostwald ripening can be completely inhibited by cellulose particles (Figure 8). This phenomenon also proved the result in Figure 6 because no significant flocculation was observed for the three emulsion droplets after storage for 9 days.

### Analysis of fat digestibility of curcumin emulsion

**Figure 9:**
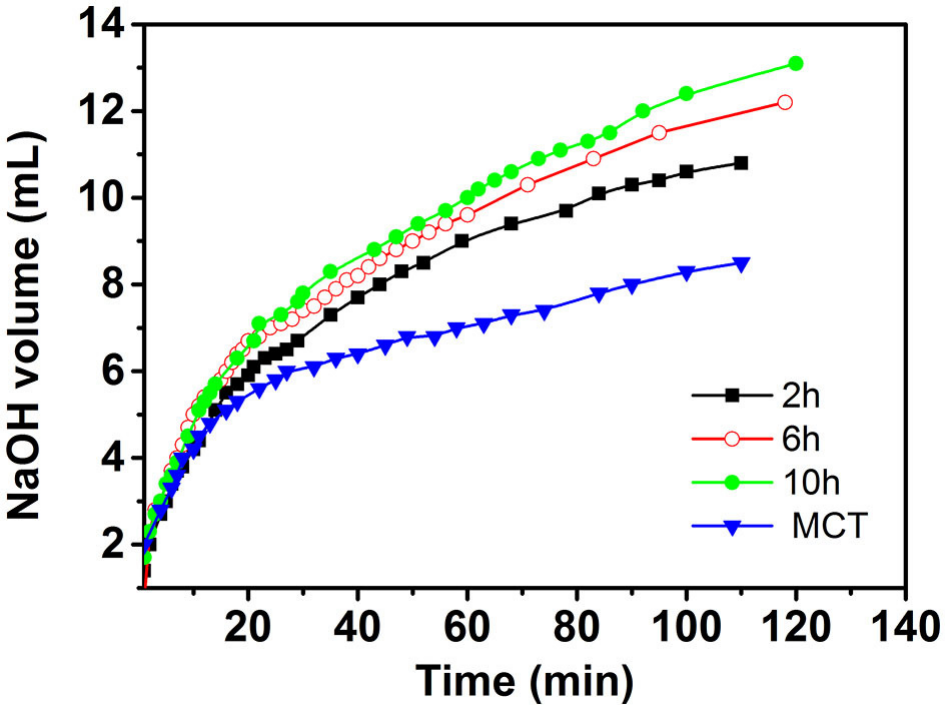
NaOH consumption of different curcumin emulsions in simulated intestinal digestion.

The *in vitro* digestion process was used to study the potential value of curcumin emulsion as a nutrient delivery system. A series of oil-in-water emulsions containing curcumin in the lipid phase was produced using the MCT and then passed through the intestinal phases of the digestion model. The kinetics of lipid digestion of the resulting samples was then monitored by measuring the amount of alkali that had to be titrated into the digestion mixture to maintain neutral pH after the addition of simulated small intestinal fluid. As shown in Figure 9, the amount of free fatty acids released from the emulsion increased sharply in the initial stage of the reaction and then increased gradually at a slower rate. This process lasts for a long time. These results indicated that free fatty acids were produced in the emulsion under the action of lipase, which was produced by the decomposition of triacylglycerols (22). However, there were statistically significant differences between the rate and extent of digestion depending on the initial degree of dispersion of the oil droplets. The total amount of alkali required to neutralize the pure MCT was lower than that required to neutralize the curcumin emulsions. These results suggested that lipid digestion was an interfacial phenomenon, and the oils in cellulose emulsion had a larger surface area, which benefited the interaction with lipase, whereas, for the bulk lipids insoluble in the water phase, the reaction between the enzyme and the oil phase would be delayed, which further decreased the free fatty acids released from lipids (23). In the case of the emulsions, the amount of alkali required for emulsion stabilized by CP6 and CP10 was higher than that of CP2. This was mainly because the emulsion stabilized by CP6 and CP10 had a smaller particle size than that of CP2, and lipase molecules rapidly attached to the surface of the lipid droplets and promoted triacylglycerol digestion (15). This result was consistent with the previous study that a smaller emulsion had a faster lipid digestion rate (24).

### Encapsulation efficiency of curcumin in emulsion

**Figure 10:**
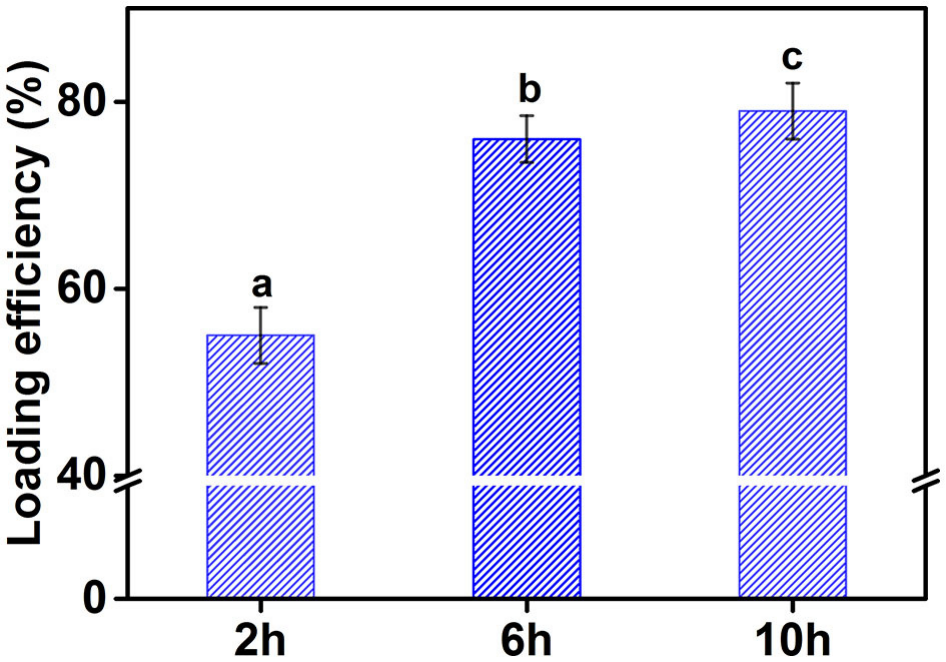
The encapsulation efficiency of curcumin in emulsion stabilized by different CP. Different characters represent the significant difference at *p* < 0.05 level (*n* = 3).

Curcumin usually exists in crystalline form, which has the disadvantages of low oral bioavailability and difficult absorption. To overcome these limitations, curcumin is usually embedded in vehicles. Commonly used carriers include delivery systems based on oil in water emulsion. This system can improve the bioavailability, dispersibility, and stability of curcumin, and is an effective delivery method. Figure 10 shows the effect of cellulose particles with different hydrolysis times on encapsulation efficiency of curcumin in an emulsion. The encapsulation efficiency of curcumin in emulsion stabilized by CP2 was about 55%, which significantly lower than that of the emulsions stabilized by CP6 and CP10. The encapsulation efficiency of curcumin significantly increased with increasing the hydrolysis time and reached 80% at 10 h. This was mainly because the emulsifying property of CP2 was poorer than that of CP10. At the same cellulose concentration, the oil phases may not be completely covered by CP2, resulting in a partial loss of curcumin. This proved once again that the emulsifying efficiency of cellulose particles increased by the decreasing the particle size. In a word, the CP obtained by hydrolysis can be used to prepare emulsion and transport curcumin and other active substances.

### Antioxidant properties of curcumin emulsion

**Figure 11:**
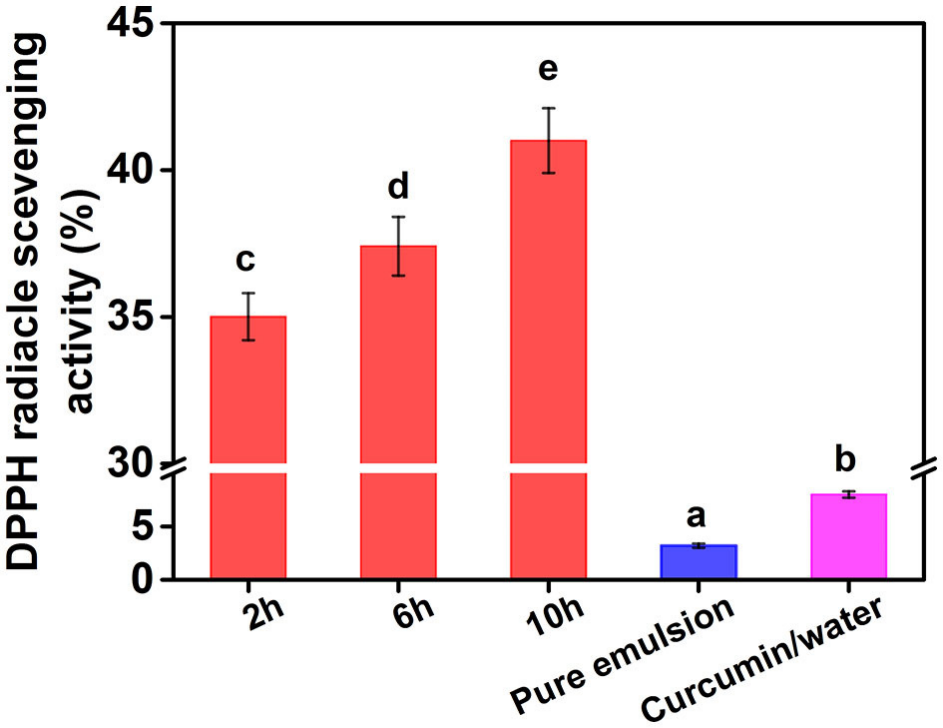
DPPH· scavenging activity of curcumin emulsion, pure emulsion, and curcumin/water. Different characters represent the significant difference at *p* < 0.05 level (*n* = 3).

The DPPH method is widely used to evaluate free radical scavenging capacity and total antioxidant capacity. After DPPH reacts with a free radical quencher, dark purple of the solution changes to colorless or light yellow (25). Curcumin has a strong antioxidant capacity and can effectively remove DPPH and other free radicals. However, it is important to maintain the antioxidant capacity of curcumin emulsion and effectively release curcumin to reduce free radicals (26). To investigate the functional properties of curcumin emulsion, the DPPH radical scavenging activity of curcumin emulsion was studied (Figure 11). At the same time, pure emulsion stabilized by CP10 was also employed to exclude the possible influence of cellulose on the result. DPPH radical scavenging activity of pure emulsion and curcumin/water was about 2.6 and 7.8%, respectively, which was much lower than that of curcumin emulsion. This was mainly because the solubility of curcumin in cellulose emulsion increased by about 1,455 times compared with curcumin/water solution (11 ng/ml). Meanwhile, DPPH scavenging activity of curcumin emulsion stabilized by CP significantly increased with increasing the hydrolysis time. These results indicated that CP with better interfacial adsorption properties could form more stable emulsions and increase the availability of active substances. Although the solubility of curcumin in emulsion was 1,455 times higher than that in aqueous solution, the DPPH· scavenging ability was only several times that of the aqueous solution. This may be because of the presence of many suspended undissolved curcumin particles in an aqueous solution that did not completely precipitate due to the short time. When Niu et al. (16) used ultra-long stable ovalbumin/carboxymethyl cellulose nanoparticles loaded with curcumin, the solubility of curcumin loaded with nanoparticles increased by about 21,000 times than that of curcumin / aqueous solution, while the DPPH· scavenging ability increased by about 30 times. Therefore, the result showed that curcumin emulsion can not only greatly improve the dispersion and stability of curcumin, but also significantly improve its functional properties.

## Conclusion

The curcumin emulsions covered with CP with different structures were successfully prepared, and the structural characteristics, stability, and antioxidant properties of emulsions were investigated. The experimental results showed that the CP obtained with increasing the hydrolysis duration had smaller particle size, better water dispersion, and interfacial adsorption capacity. The encapsulation efficiency of curcumin in emulsion significantly increased and reached about 80% at 10 h. The mean particle size of curcumin emulsions stabilized by CP2 was significantly higher than that of CP6 and CP10. As the CP size decreases, the curcumin emulsion also became more stable. After 9 days, all emulsions showed good stability, and no obvious creamed layer was observed. The amount of alkali required for emulsion stabilized by CP6 and CP10 was higher than that of CP2, suggesting that smaller emulsion droplets had a faster lipid digestion rate. Meanwhile, DPPH scavenging activity of curcumin emulsion stabilized by CP significantly increased on increasing the hydrolysis duration, which was much higher than that of pure emulsion and curcumin/water. This was mainly because the solubility of curcumin in cellulose emulsion increased by about 1,455 times compared with the curcumin/water solution. Thus, these results indicated that CP emulsion could improve not only the stability and dispersion of curcumin, but also its functional properties. However, this study evaluated the antioxidant properties of encapsulated curcumin *in vitro*, and animal models should be selected to further study the functional properties of curcumin *in vivo*.

## Data availability statement

The original contributions presented in the study are included in the article/supplementary material, further inquiries can be directed to the corresponding authors.

## Author contributions

All authors listed have made a substantial, direct, and intellectual contribution to the work and approved it for publication.

## Funding

This research was supported by Zhejiang Provincial Top Discipline of Biological Engineering (Level A) (No. ZS2021009), Zhejiang Provincial Top Discipline of Biological Engineering (Level A) open fund (Nos. KF2020004 and KF2021011), and 2020 Ningbo Public Welfare Science and Technology Plan Project (No. 202002N3098).

## Conflict of interest

The authors declare that the research was conducted in the absence of any commercial or financial relationships that could be construed as a potential conflict of interest.

## Publisher's note

All claims expressed in this article are solely those of the authors and do not necessarily represent those of their affiliated organizations, or those of the publisher, the editors and the reviewers. Any product that may be evaluated in this article, or claim that may be made by its manufacturer, is not guaranteed or endorsed by the publisher.
