# Supplementary figures and images for: Emulsions stabilized by cellulose-based nanoparticles for curcumin encapsulations: In vitro antioxidant properties
Source: Front Nutr. 2022 Jul 22;9:931581. doi: 10.3389/fnut.2022.931581 (PMC9356219; doi:10.3389/fnut.2022.931581)

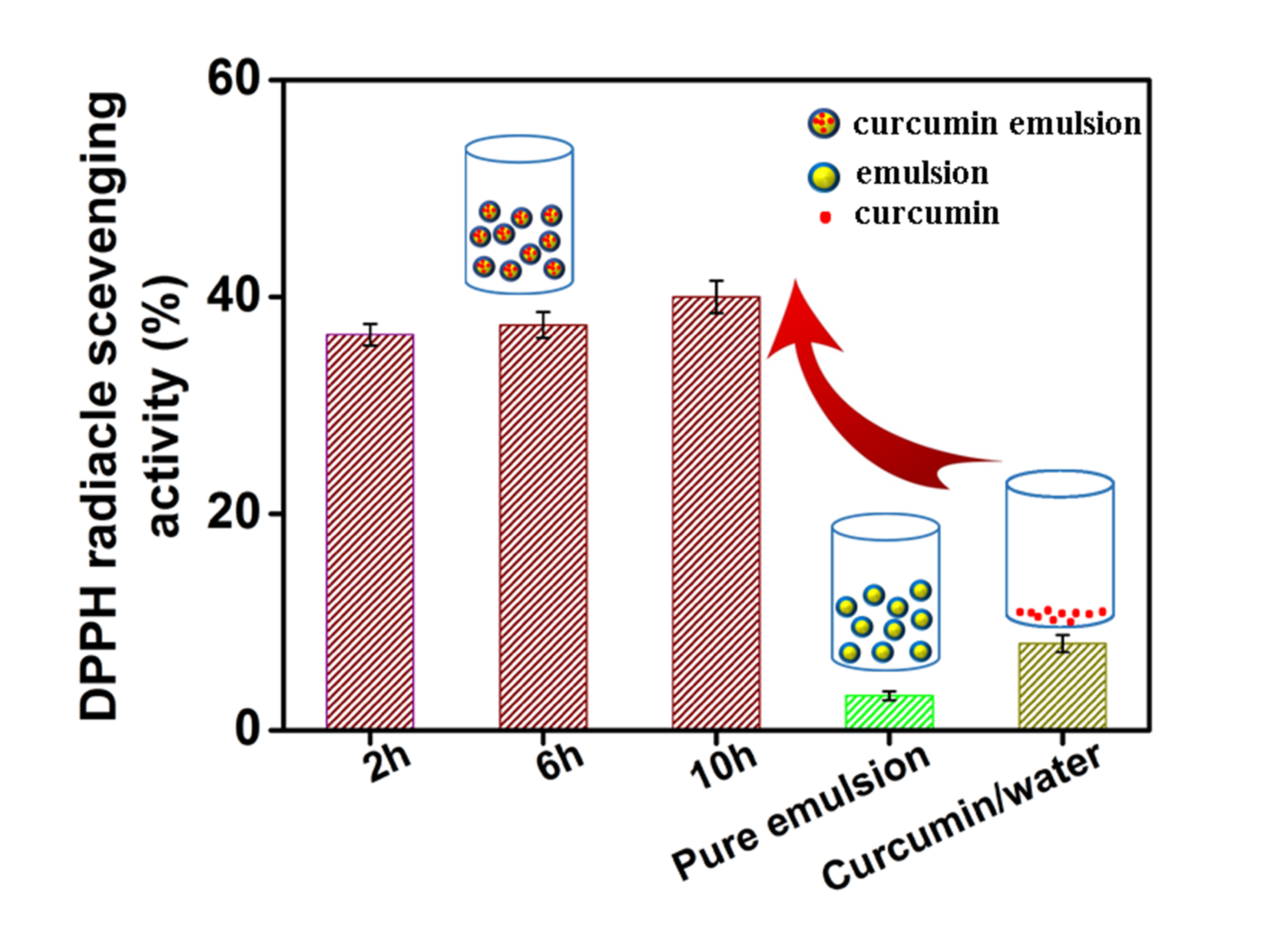

Supplement: Supplementary file 1 [file Image_1.TIF]
